# The Importance of Molecular Size, Concentration, and Thermal Conditions in Enhancing Lignin Derivatives’ Interactions with Skin-like Membranes: Implications for Cosmetic and Therapeutic Applications

**DOI:** 10.3390/ijms26209906

**Published:** 2025-10-11

**Authors:** Alexandra Farcas, Alex-Adrian Farcas, Lorant Janosi

**Affiliations:** 1 National Institute for Research and Development of Isotopic and Molecular Technologies, 67-103 Donat, 400293 Cluj-Napoca, Romania; alexandra.farcas@itim-cj.ro (A.F.); alex.farcas@itim-cj.ro (A.-A.F.); 2 Department of Physics and Chemistry, Technical University of Cluj-Napoca, 400641 Cluj-Napoca, Romania

**Keywords:** lignin, cosmetic applications, skin membranes, molecular dynamics, free energy calculations

## Abstract

Lignin is one of the most abundant natural biopolymers and plays a crucial role in the development of safe and sustainable alternatives for healthcare products. In this study, we employed molecular dynamics simulations and free energy calculations to investigate lignin derivatives’ interactions with skin-like membranes. Specifically, we designed a small lignin derivative composed of syringyl and guaiacyl subunits. Our results reveal that molecular size, concentration, and thermal conditions critically influence the insertion, interaction dynamics, and localization behavior of lignin derivatives. Notably, variations in these parameters induce distinct behaviors, including rapid membrane insertion, hydrogen bonding, clustering, and surface adhesion. The findings provide insights into the molecular mechanisms governing lignin derivatives’ interactions with skin-like membranes, with implications for developing bio-based skincare formulations and transdermal delivery systems. Our results highlight the importance of molecular size and concentration in optimizing lignin-derived compounds for dermatological and therapeutic applications.

## 1. Introduction

In recent years, there has been a growing demand for the development of natural products suitable for use in various industries due to their eco-friendly and sustainable nature [1,2,3,4]. This demand has spurred significant research into natural biopolymers derived from renewable resources, with the potential to replace or complement synthetic compounds currently used across these sectors [5]. Among these biopolymers, lignin—an abundant and complex aromatic polymer found in plant cell walls—has gained considerable attention for its potential applications in a range of fields, including the medical, cosmetic, pharmaceutical, agricultural, packaging, biorefinery, construction, and textile industries [6,7,8,9,10]. Estimated to exist in around 300 billion tonnes within the biosphere, lignin includes 50 to 70 million tonnes sourced from black liquor produced by the paper and pulp industry [11]. Despite a global production capacity of approximately 265,000 tonnes per year, the majority of lignin is currently incinerated as low-cost fuel, and only about 2% is utilized in the manufacturing of chemicals and materials such as dispersants, adhesives, and surfactants [12,13,14,15,16]. This significant underutilization underscores the opportunity to explore value-added applications for lignin, particularly in promoting sustainable practices and reducing reliance on synthetic materials.

In the medical sector, lignin’s biocompatibility makes it a promising candidate for drug delivery systems, where its ability to form hydrogels allows for effective encapsulation and controlled release of pharmaceuticals [17,18]. Furthermore, lignin’s antioxidant properties contribute to the development of wound healing materials and skin regeneration therapies [19]. The cosmetic industry benefits from lignin’s natural antimicrobial and anti-inflammatory characteristics, enhancing the efficacy of skincare formulations and improving their appeal to consumers [20,21,22]. Additionally, lignin’s role as a natural emulsifier adds value to creams and lotions by contributing to their texture and stability while ensuring environmental safety [6,7]. Given the projected growth of the lignin market, which is expected to exceed USD 1.5 billion by 2025 with growth rates of over 12% annually [14], this demonstrates the pressing need to investigate lignin’s applications further, particularly in cosmetic and therapeutic sectors, in order to fully harness its vast potential and address the growing demand for sustainable products.

**Figure 1:**
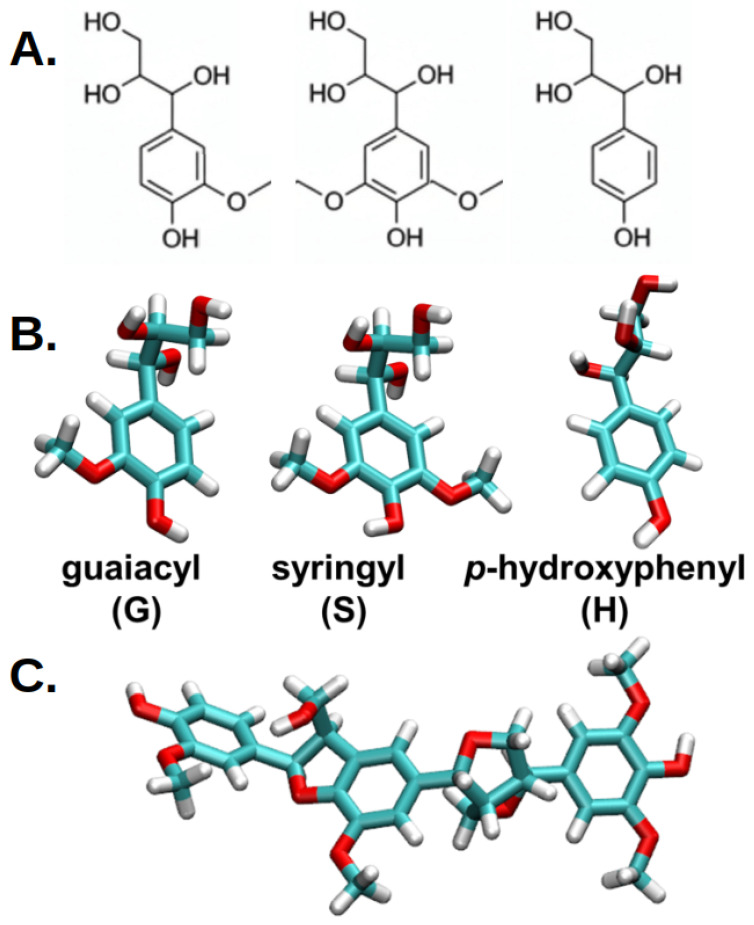
(**A**) illustrates the chemical structures of the fundamental building blocks of lignin—namely, the guaiacyl (G), syringyl (S) and p-hydroxyphenyl (H) subunits [18]. (**B**) provides a detailed visual representation of the molecular structures involved in lignin modeling, highlighting key subunits and their composition. These subunits are essential components of lignin’s complex polymeric network, with guaiacyl units characterized by their methoxy groups attached to the aromatic ring, and syringyl units distinguished by their additional methoxy substituents. The carbon atoms within these subunits are depicted in cyan, allowing for clear visualization of the aromatic backbone, while oxygen atoms are highlighted in red, emphasizing the sites of potential chemical interactions or modifications and hydrogen atoms in white. (**C**) showcases a lignin derivative used in computational simulations, composed of two guaiacyl subunits and one syringyl subunit.

Lignin is the second most abundant natural polymer on Earth after cellulose, constituting a significant portion of the plant cell wall. Lignin is primarily composed of three types of monolignol units: guaiacyl (G), syringyl (S), and p-hydroxyphenyl (H) units (see Figure 1). These monolignol units serve as precursors to the lignin polymer structure, contributing to its complex network and unique properties. Guaiacyl units are derived from guaiacyl alcohol, syringyl units from syringyl alcohol, and p-hydroxyphenyl units originate from p-coumaryl alcohol. The specific ratios and arrangements of these units in lignin can vary significantly depending on the plant species, influencing lignin’s functionality and reactivity in various applications [23,24]. Lignin’s high molecular weight and intricate structure contribute to its resilience and protective roles in plants, including shielding cellulose from microbial degradation [25]. These properties, along with its high availability as a byproduct of industrial processes such as papermaking, have made lignin and its derivatives attractive candidates for a wide array of biomedical applications, from drug delivery systems to wound healing and skin care formulations [8,26]. Indeed, the antioxidant and antibacterial properties of lignin derivatives, particularly its phenolic components, have led to investigations into their therapeutic potential for treating various diseases, including diabetes [27], cancer [28], and HIV [29,30].

In particular, the potential of lignin nanoparticles as drug delivery vehicles has garnered significant interest [31,32]. Lignin’s ability to encapsulate hydrophobic drugs and its non-toxic, biocompatible nature make it an ideal candidate for targeted drug delivery systems [33,34]. The ability of lignin derivatives to form nanoparticles with controlled size and surface charge further enhances their potential for drug delivery, allowing for better bioavailability and controlled release [31]. Lignin nanoparticles function as a UV-protective drug delivery platform that stabilizes and ferries thymine-based photo-adducts to the skin, enabling controlled release and subsequent antimelanoma activity [35]. However, the interaction between lignin derivatives and biological membranes, particularly those of the skin, remains a topic of significant research interest [36,37]. Lignin’s amphiphilic nature, meaning it possesses both hydrophilic and hydrophobic properties, makes it an ideal candidate for interacting with the lipid bilayer structure of skin membranes, facilitating enhanced permeability and absorption of other active substances [38,39,40].

Recent studies have highlighted lignin’s ability to disrupt skin membranes composed of lipids [41,42]. Lignin interacts with skin lipid bilayers, leading to increased membrane fluidity and permeability, which could improve the skin’s ability to absorb other therapeutic or cosmetic agents [43]. Its antioxidant properties demonstrate protective effects against oxidative stress in skin cells, which are often caused by exposure to environmental stressors such as UV radiation and pollution [44]. Additionally, lignin has been shown to possess anti-inflammatory properties, with studies indicating its ability to inhibit the production of pro-inflammatory cytokines in skin cells, suggesting its potential for use in anti-aging and anti-inflammatory skincare formulations [45,46].

Despite these promising findings, several challenges remain in the commercialization and effective application of lignin-based products [47,48]. The inherent recalcitrance of lignin makes it challenging to modify its chemical structure for optimal compatibility with biological systems [49,50]. Its poor thermoplasticity further restricts its processability, limiting its use in applications that require versatile polymer behaviors, such as in emulsions or carrier systems for topical formulations [51,52]. Another major hurdle is the lack of comprehensive understanding regarding the interaction mechanisms between lignin derivatives and biological membranes, especially skin membranes [37]. While lignin has been shown to interact with lipid bilayers, the specific functional groups responsible for these interactions are not well characterized [43]. Moreover, there is a limited body of scientific research [37] focused on exploring the behavior of lignin and its derivatives when exposed to model skin membranes, a critical aspect for developing effective skincare products.

Ceramides are essential sphingolipids that play a pivotal role in maintaining the integrity and functionality of skin membranes [53]. Found in the stratum corneum, the outermost layer of the skin, ceramides contribute significantly to the formation of the lipid bilayer, which serves as a barrier to prevent transepidermal water loss and external environmental aggressors [54]. Their unique structure enables them to form strong hydrogen bonds with neighboring lipids, promoting the stability of the skin barrier and enhancing its resilience against stressors such as dehydration and irritation [55,56]. Moreover, ceramides are crucial for signaling processes that are involved in skin repair and inflammation, illustrating their dual role in both structural integrity and biological function [57]. Given these key characteristics, the incorporation of ceramides into models of skin-like membranes is essential for studying the interactions of lignin derivatives with components that mimic the natural skin environment. This understanding of ceramide dynamics in skin membranes is crucial for developing effective cosmetic and therapeutic applications, as it allows for optimizing formulations that utilize lignin derivatives to enhance skin health and barrier function [58]. By investigating these interactions, we can pave the way for innovative products that leverage the unique benefits of lignin while ensuring compatibility with key skin components like ceramides [3].

Therefore, understanding the interaction of lignin derivatives (see Figure 1) with model skin membranes is crucial for advancing the application of lignin in cosmetic and pharmaceutical industries. By studying these interactions in detail, we will not only gain insights into the molecular mechanisms underlying the absorption and skin penetration of lignin derivatives but also identify the potential for creating innovative, safe, and sustainable products that integrate natural components into these industries. Moreover, such advancements could facilitate the large-scale production of lignin-based materials, paving the way for their inclusion in a wide variety of cosmetic and medical applications. This article aims to explore how molecular size, concentration, and thermal conditions influence the interactions of lignin derivatives with skin-like membranes, thereby optimizing their potential for cosmetic and therapeutic applications.

## 2. Results

We analyzed molecular dynamics (MD) trajectories to examine the guaiacyl and syringyl units, as well as representative lignin derivatives, within a ceramide bilayer system. Our primary derivative model was a trimer consisting of guaiacyl–guaiacyl–syringyl residues, chosen for its structural relevance in capturing the essential interactions between guaiacyl and syringyl subunits. This model allowed us to probe the behavior of lignin derivatives within lipid membranes and assess their influence on the ceramide bilayer structure. The chemical composition and structure of this lignin trimer are illustrated in Figure 1. We selected the trimer model because it offers a clear, interpretable platform to illustrate how unit composition and mixing influence properties at the molecular level, thereby informing design principles for more complex lignin-based systems. This controlled framework enables systematic investigation of how lignin motifs influence UV absorption and stability, consistent with evidence that lignin-rich materials exhibit enhanced UV-filtering at lower molecular weights [59]. As a proof of concept, we show that mixing lignin units modulates properties, enabling the development of mixed/engineered lignin derivatives customized for specific skin-membrane environments. In total, we performed over 6 µs of MD simulations, including a 300 ns control simulation of the ceramide bilayer without lignin derivatives to establish baseline structural and dynamic properties (see Table 1).

### 2.1. Surface Adhesion of Lignin

**Figure 2:**
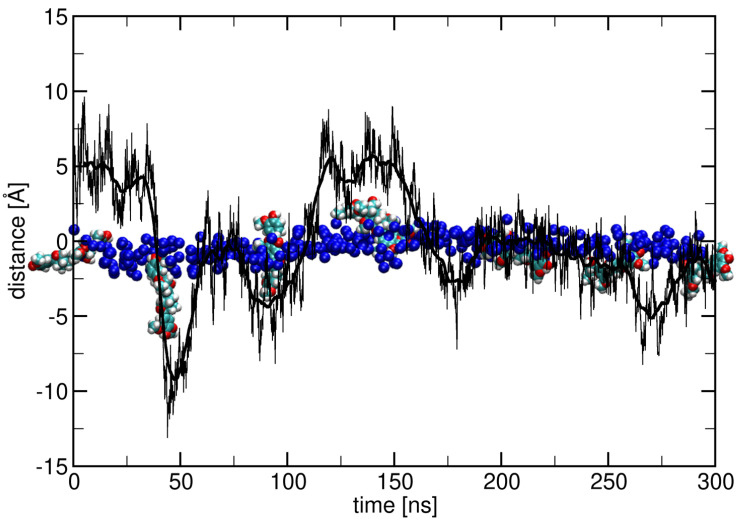
The distance was calculated as the difference between the average z coordinates of the nitrogen atoms of the ceramide layer and the center of mass of the lignin derivative. The inset shows the lignin derivatives in vdW representation, with cyan-colored carbon atoms, red oxygen atoms, white hydrogen atoms, and nitrogen atoms of the ceramide layer represented as blue spheres. The smooth black distance curve was obtained by running average over 10 ns.

**Figure 3:**
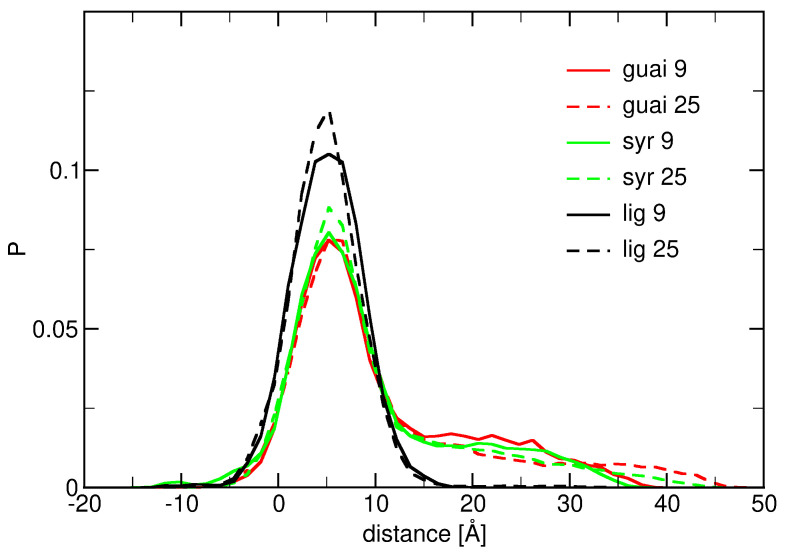
Distribution of COM distances of the guaiacyl units (red), syringyl units (green), and lignin derivatives (black) to the membrane at T = 310 K. The distance was calculated as the difference between the average z-coordinate of the nitrogen atoms and the center of mass of the guaiacyl unit, the syringyl unit, and the lignin derivative. Solid lines correspond to a concentration of 9 molecules, and dashed lines represent 25 molecules of each compound.

The surface adhesion of lignin derivatives constitutes an initial step that governs their subsequent interactions with skin-like membranes. To elucidate how this occurs, we examined the spatial distribution of lignin derivatives relative to the lipid bilayer by measuring the distance along the membrane normal (z-axis) between their centers of mass and the average position of nitrogen atoms in the upper leaflet of the membrane. These measurements, presented in Figure 2 and Figure 3, reveal that lignin derivatives rapidly associate with the membrane surface across all simulation conditions. This binding behavior indicates a strong affinity of the lignin derivatives to the ceramide bilayer, facilitating their potential incorporation into the lipid membrane structure.

The molecular behavior of lignin derivatives interacting with ceramide bilayers has been investigated by simulating systems at three different temperatures: 300 K, 310 K, and 320 K (see Figure 3, Figure A6 and Figure A7). These simulations aimed to explore the normalized distributions of the distance between the nitrogen atoms of the ceramide bilayer and the center of mass (COM) of guaiacyl units, syringyl units, and lignin derivatives at two different concentrations. The solid lines in the figures represent systems with 9 molecules, while the dashed lines correspond to systems with 25 molecules of each compound. The results from these simulations provide insights into the interaction dynamics of lignin derivatives with the lipid membrane.

At T = 300 K (see Figure A6), the distributions of guaiacyl and syringyl units exhibit relatively distinct features. The overall distribution profiles at this temperature indicate a preference for a relatively stable interaction between the lignin derivatives and the membrane. Specifically, the distributions for guaiacyl and syringyl units display two peaks, which symbolize that some molecules are dispersed in water while others adhere to the bilayer. The lignin derivatives demonstrate a narrow distribution profile, suggesting that sampling predominantly occurs at the interface between water and the ceramide bilayer. This behavior can be attributed to the larger size and more complex structure of the lignin derivatives, which influence their interaction and localization within the membrane environment. Increasing the concentration of lignin derivatives from 9 to 25 molecules results in a narrowing of their distribution profile. This observation suggests that higher concentrations promote more ordered packing of these molecules within the bilayer, potentially leading to more stable and homogeneous interactions with the membrane. At elevated temperatures of 310 K (see Figure A6) and 320 K (see Figure A7), the distribution profiles for guaiacyl, syringyl units, and lignin derivatives remain largely unchanged. This temperature invariance indicates that the interaction characteristics of lignin derivatives with the membrane are maintained across this temperature range. Such stability supports the suitability of lignin derivatives for incorporation into cosmetic formulations, as the structural and functional integrity of skin-mimicking membranes is preserved under these thermal conditions.

Furthermore, the behavior of lignin derivatives markedly differs from that of guaiacyl and syringyl units. While the latter exhibit consistent presence both in water and at the membrane interface across all tested temperatures (see Figure 3 and Figure A7), lignin derivatives display narrow distributions, particularly at higher concentrations. This suggests a greater propensity for lignin derivatives to aggregate or form complex interactions within the bilayer environment. Lignin derivatives demonstrate rapid binding to the ceramide bilayer across all examined temperatures and concentrations. However, this interaction is sensitive to molecular structure and concentration, with elevated concentrations fostering stronger associations. These findings elucidate the nuanced behavior of lignin derivatives within lipid bilayer systems and underscore their potential to modulate membrane properties. Such insights are critical for assessing their role in biological and bioengineering applications, where they may affect membrane stability, permeability, and overall function, especially under conditions of elevated temperature.

Figure A2 illustrates the number density profiles of various atoms across the ceramide bilayer–water interface, plotted as a function of the distance from the bilayer center. The cyan curve labeled “water” dominates in the regions far from the bilayer center (|distance| > 20 Å), corresponding to the bulk aqueous phase. Its steep drop near ±20 Åmarks the water–bilayer interface. Inside the bilayer, multiple atomic species contribute to the density: O1, NF, C9F, C9S, C18S, and C18F. These species exhibit distinct peaks at specific distances, indicating preferential locations within the bilayer structure. For example, O1 and NF peaks occur closer to the interface, reflecting their association with polar headgroups, while C9F, C9S, C18S, and C18F peaks are more central, consistent with their hydrocarbon chain locations. The symmetry around the bilayer center reflects the bidirectional orientation of ceramide molecules in each leaflet. Overall, this density profile offers insight into the spatial organization of ceramide atoms relative to water.

Figure A3 focuses on the number density distributions of water, the ceramide bilayer, and lignin derivatives. Water (cyan) maintains a high density in the bulk regions beyond ±20 Å, sharply decreasing as it approaches the bilayer surface. The brown curve represents the entire ceramide bilayer, showing strong, broad peaks between approximately −20 Å and +20 Å, indicating the dense, hydrophobic lipid core. The lignin derivatives, shown in green, are present in smaller quantities but demonstrate a noticeable enrichment at the bilayer surface at around ±20 Å. Their density does not extend deeply into the hydrophobic core, implying that these molecules remain primarily at the interfacial zone, most likely due to their amphiphilic nature. Nonetheless, these number density distributions also highlight that the lignin derivatives have limited penetration under these conditions.

**Figure 4:**
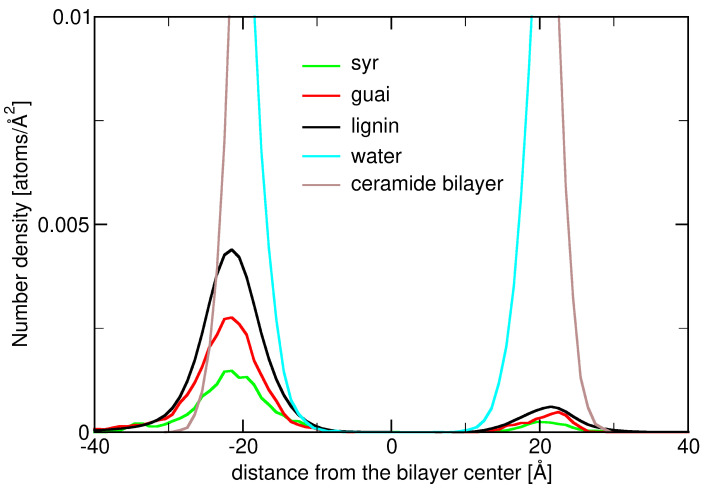
Number density profiles of lignin derivatives (black) and their two components, syringyl (green) and guaiacyl (red), together with water (cyan) and the ceramide bilayer (brown) at T = 300 K, for a system containing 25 lignin derivatives (zoom in from Figure A3).

Figure 4 expands upon the previous analysis decomposing the lignin derivative density into its constituent monomeric units: syringyl (Syr, green), guaiacyl (Guai, red), and the total lignin density (black). The lignin derivative and its three subunits (two guaiacyls and one syringyl) exhibit very similar number density plots. Their peak positions (around 21.5 Å), their range, and widths at half maximum are fairly the same. Since lignin derivative’s distribution is the sum of the other two (they are one molecule), one can conclude that, at the interfacial region, the lignin derivative is lying most of the time with its components (two guaiacyls and one syringyl) aligned perpendicular to the ceramide membrane’s normal. These results point to a preferential interaction of lignin monomers with the ceramide–water interface, possibly driven by aromatic–hydrophobic and hydrogen-bonding interactions, while still being restricted from the hydrophobic bilayer interior.

Unbiased molecular dynamics simulations of guaiacyl, syringyl, and a lignin derivative (composed of two guaiacyls and one syringyl units) in interaction with a fully solvated ceramide bilayer have shown that lignin molecules are mostly present in the close vicinity of the ceramide bilayer. Furthermore, short-lived spontaneous insertions up to medium depths (half way into the ceramide bilayer) do occur without bias, suggesting that the subunits and especially the lignin derivative are prone not only to interfacial interaction with the ceramide bilayer but also to insertion.

### 2.2. Influence of Hydration on Lignin–Membrane Interactions

**Figure 5:**
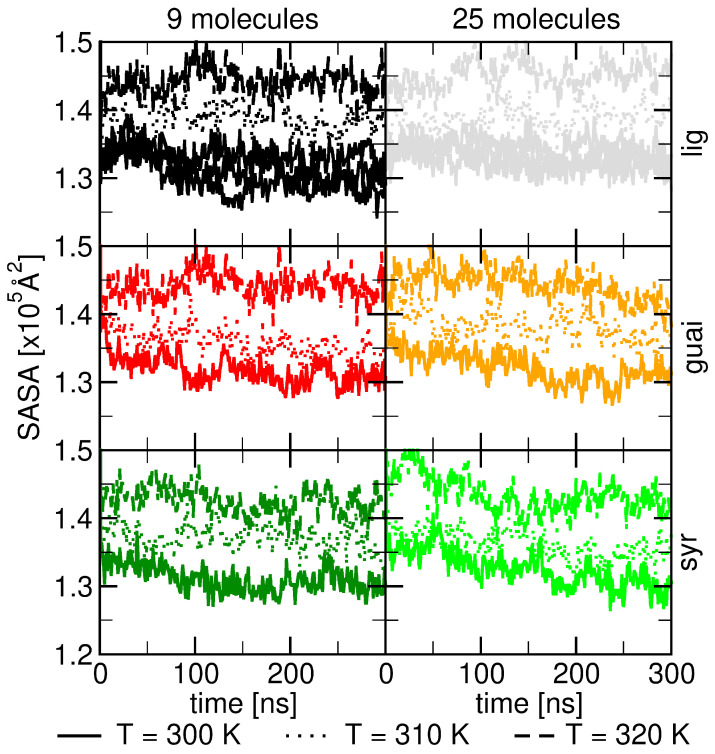
Solvent-accessible surface area (SASA) of ceramide bilayers from MD simulations in the presence of guaiacyl units (red/orange), syringyl units (dark/light green), and lignin derivatives (black/grey). Each molecule is represented in a different row. The systems contain two concentrations (9 and 25 molecules), with each concentration in a separate column. Data are shown across different temperatures (300, 310, and 320 K), depicted with different line styles.

Based on our simulations investigating lignin–lipid interactions, the solvent-accessible surface area (SASA) profiles shown in the figure reveal how temperature and molecular concentration influence the behavior of lignin derivatives within ceramide bilayers. The SASA data, collected at three different temperatures (300 K, 310 K, and 320 K), highlight that increasing temperature consistently enhances the solvent exposure of lignin derivatives. At higher temperatures, the lignin molecules, including guaiacyl, syringyl, and other lignin units, exhibit greater flexibility and exposure to the surrounding solvent, as indicated by the rising SASA values (see Figure 5).

At the higher concentration (25 molecules), the SASA of the ceramide bilayer is is a little higher compared to the lower concentration (9 molecules), especially at elevated temperatures (310 K and 320 K). The higher molecular density leads to more interactions between the lignin derivatives and the ceramide bilayer, which enhances the surface exposure of the bilayer to the solvent (see Figure 5).

The observed order of increasing solvent-accessible surface area (SASA) among guaiacyl units, syringyl units, and the full lignin derivative can be attributed to their differing interactions with the ceramide bilayer. Guaiacyl units exhibit a higher hydrogen-bonding capacity due to their stronger interactions with water, which keeps them more embedded within the hydrophobic core, resulting in the lowest SASA (see Figure A9). In contrast, syringyl units contain two methoxy (–OCH_3_) groups that sterically hinder water access to the hydroxyl group, reducing their hydrogen-bonding potential relative to guaiacyl units [60]. The lignin derivative, composed of two guaiacyl units and one syringyl unit, therefore shows an intermediate behavior.

**Figure 6:**
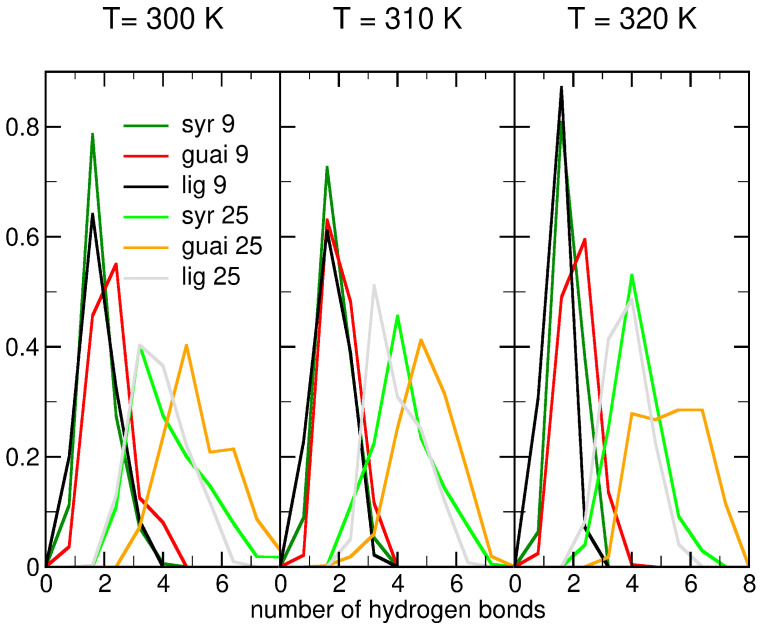
Normalized distribution of the number of intermolecular hydrogen bonds between lignin molecules and the ceramide bilayer at temperatures of 300 K, 310 K, and 320 K in each column, respectively. The systems consist of ceramide bilayers with 9/25 lignin molecules of syringyl units (dark/light green lines), guaiacyl units (red/orange lines), and lignin derivatives (black/grey lines), respectively. At lower concentrations (darker lines), no significant differences are observed; however, as the concentration increases, guaiacyl units form more hydrogen bonds than syringyl units and other lignin derivatives at all temperatures, facilitating easier insertion for lignin derivatives.

Our molecular dynamics simulations further indicate that temperature and molecular concentration amplify these effects: higher temperatures increase molecular flexibility, enhancing SASA for all lignin types, and higher concentrations promote additional lignin–bilayer interactions, slightly raising the overall SASA (see Figure 5 and Figure 6). Therefore, both structural features and dynamic molecular behavior explain the slight gradual increase in SASA in the order guaiacyl < syringyl < lignin derivative. Hence, MD simulations show a slight increase in SASA from guaiacyl units and syringyl units to lignin derivatives. Such behavior is similarly found with increasing lignin concentration. However, a significant change was found in SASA when steadily increasing temperature from 300 K to 320 K.

In Figure A4, we present the number density distributions of water at varying temperatures, T = 300 K, T = 310 K, and T = 320 K, for systems containing syringyl and guaiacyl units, as well as lignin derivatives. We observe that with increasing temperature, water penetrates more deeply into the ceramide bilayer, in accordance with the literature [56,61]. The influence of relative humidity is indeed an important factor, as higher humidity levels can enhance water interactions and increase the plasticization of both lignin [62] and ceramide membranes [56]. This effect lead to alterations in the structural and dynamic properties of these molecules, potentially modifying their interaction mechanisms within the bilayer environment [56,62]. Figure A5 illustrates the solvent-accessible surface area (SASA) per molecule for syringyl and guaiacyl units, as well as lignin derivatives. Due to clustering at higher concentrations, the SASA is smaller compared to lower concentrations. Notably, we observe that the SASA of lignin derivatives is considerably (20–25%) higher in comparison with the guaiacyl and syringyl units (being slightly higher than that of guaiacyl).

### 2.3. Insertion into Skin-like Membranes

**Figure 7:**
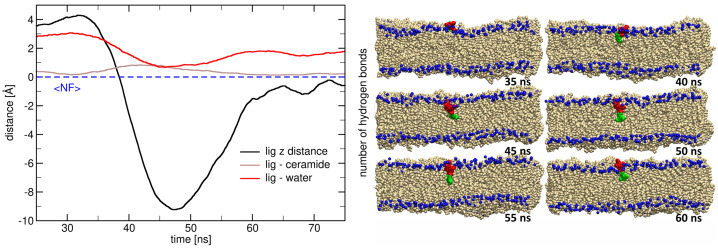
(**Left**) The plot illustrates the z-distance from the center of mass (COM) of the lignin derivative (black line) relative to the ceramide bilayers’ nitrogen atoms’ (NF) position (dashed blue line) over time. The graph also depicts the number of hydrogen bonds formed between water and the lignin derivative (red), which decreases close to zero as the lignin derivative is deeply inserted into the membrane between 40 to 50 ns. Conversely, the number of hydrogen bonds between the ceramide bilayer and the lignin derivative (brown) slightly increases as the lignin derivative inserts into the bilayer. This dynamic interplay highlights the changing interactions as the lignin derivative transitions from a more hydrated environment into the lipid membrane. (**Right**) The series of snapshots (t = 35 ns, 40 ns, 45 ns, 50 ns, 55 ns, and 60 ns) depicts the spontaneous insertion of the lignin derivative into the ceramide membrane. For clarity, water molecules and ions have been omitted from the visualization. The lipid bilayer is represented using Van der Waals (VDW) representation, with the nitrogen atoms from the surface of the ceramide bilayer shown as blue spheres. The lignin derivatives, composed of guaiacyl units shown in red and syringyl units in green, are also depicted using VDW representation. These snapshots effectively illustrate the gradual insertion of the lignin derivative into the ceramide bilayer, providing insights into the structural and interactive dynamics at play during the insertion process.

The data presented in Figure 7 reveal the dynamic process by which a lignin derivative transitions from the aqueous phase into the ceramide bilayer. The plot in the left panel shows that the z-distance of the lignin derivative’s COM to the ceramide bilayer (black line) decreases sharply from 40 ns to 50 ns (maintaining large negative values up to 60 ns), indicating membrane penetration. This movement is accompanied by a marked reduction in hydrogen bonding with water molecules, highlighting the departure of the lignin derivative from the aqueous environment. While insignificant, the number of hydrogen bonds between the lignin derivative and ceramide molecules increases as well. The complementary trends in these interaction profiles strongly support a major role of hydrogen bonding with hydration water (red line) in stabilizing the lignin derivative at the bilayer interface and a less important role of hydrogen bonds during insertion into the ceramide bilayer.

The structural snapshots (right panel) further illustrate the insertion pathway of the lignin derivative at different time points. At 35 ns, the lignin derivative remains above the bilayer surface, forming hydrogen bonds with the surrounding water molecules. Between 40 and 45 ns, the molecule begins to penetrate into the hydrophobic region, with its guaiacyl and syringyl units orienting parallel to the lipid tails. From 50 to 55 ns, the lignin derivative is fully embedded within the membrane interior, where it establishes new interactions with ceramide headgroups and lipid tails. After 60 ns, the lignin derivative starts to re-emerge from the bilayer and brake all the hydrogen bonds with the ceramide bilayer.

Overall, these results demonstrate the spontaneous ability of lignin derivatives to insert into ceramide membranes without external bias, driven largely by the redistribution of hydrogen-bonding interactions from water to lipid molecules. These unbiased membrane insertions can be explained by the amphiphilic nature of the lignin derivatives, which enables them to bridge hydrophilic and hydrophobic environments. Such behavior has implications for their potential applications in skin-related systems, where lignin derivatives may interact with stratum corneum lipids, influencing barrier properties, permeability, and hydration. The combined quantitative and structural data thus provide mechanistic insights into the molecular basis of lignin–membrane interactions.

**Figure A1:**
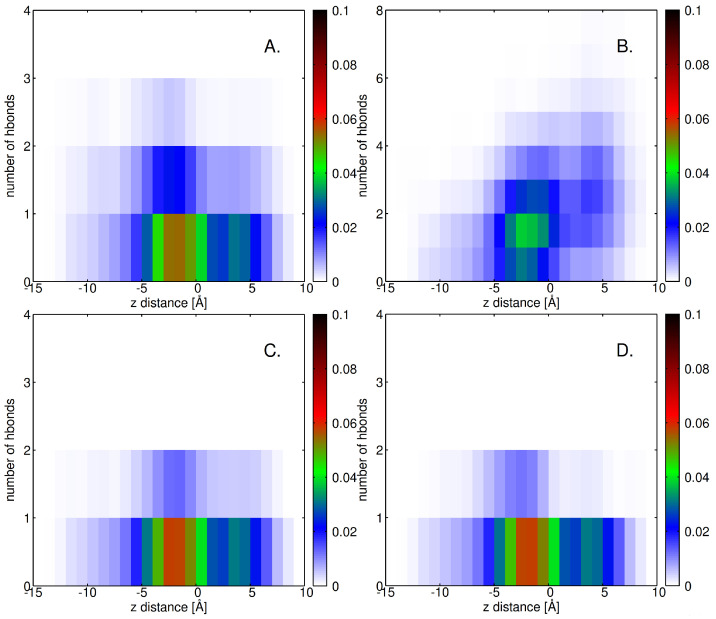

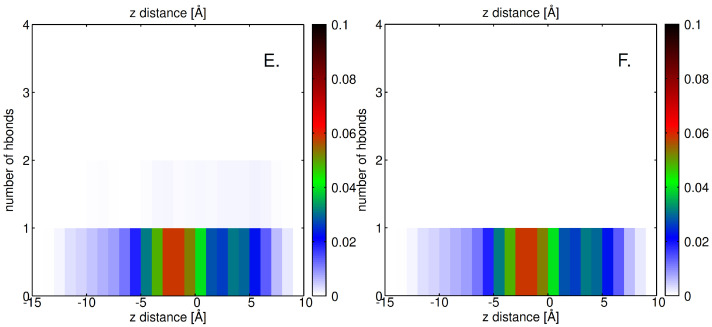
The figure presents the number of hydrogen bonds formed between the lignin derivative and various components, plotted against the *z*-distance of the lignin derivative molecule. The *z*-distance is measured from the center of mass (COM) of the lignin derivative relative to the nitrogen atoms of the ceramide bilayer (positioned at z=0). (**A**) shows the number of hydrogen bonds formed with the ceramide bilayer. (**B**) displays the number of hydrogen bonds with water molecules. (**C**) illustrates the number of hydrogen bonds with nitrogen (NF) atoms. (**D**) shows the number of hydrogen bonds with oxygen (OF) atoms. (**E**) represents the number of hydrogen bonds with oxygen 1 (O1) atoms. Finally, (**F**) depicts the number of hydrogen bonds with oxygen 3 (O3) atoms. The variation in the hydrogen bond formation across the *z*-distance provides insights into the interactions of the lignin derivative as it moves from a more aqueous environment into the lipid bilayer.

**Figure A2:**
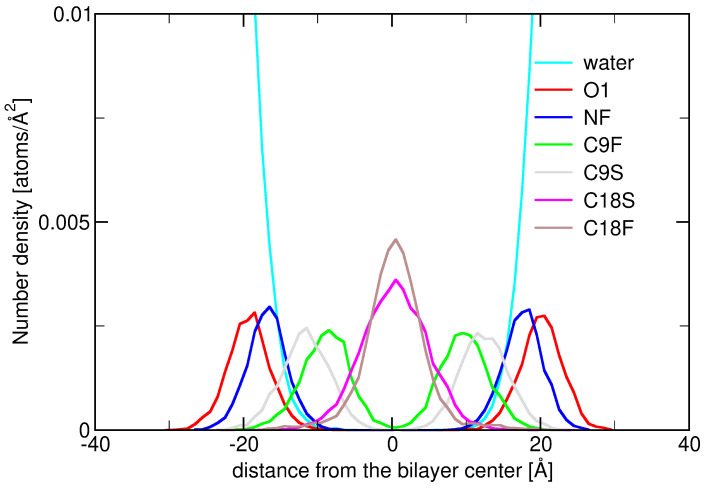
Number density profiles of various atomic groups across the ceramide bilayer as a function of the distance from the bilayer center. Inside the bilayer, distinct peaks for O1 (red), NF (blue), C9F (green), C9S (grey), C18S (magenta), and C18F (brown) indicate preferential locations: polar headgroup-associated atoms peak near the interface, while hydrocarbon chain carbons peak closer to the bilayer center. The symmetry reflects the bidirectional orientation of ceramide molecules in both leaflets.

**Figure A3:**
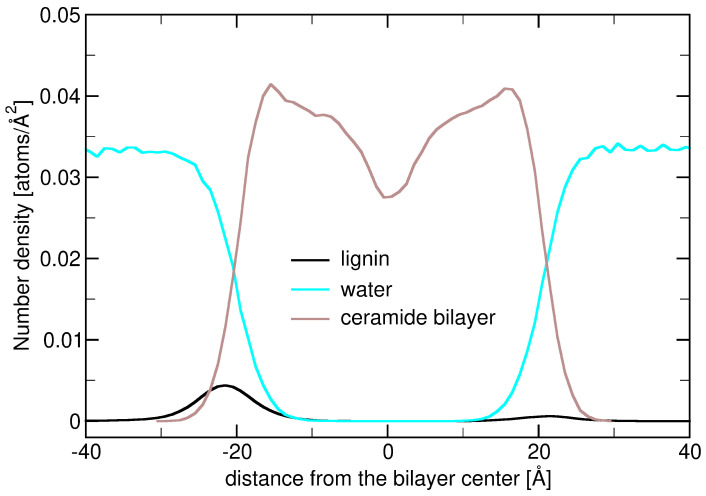
Number density distributions of water (cyan), ceramide bilayer (brown), and lignin derivatives (black) at T = 300 K. The x-axis represents the distance from the bilayer center, while the y-axis indicates the corresponding number density of each component. The sharp peak in the blue curve signifies a high concentration of water molecules densely packed at the interface, reflecting their strong interactions with the ceramide bilayer. The brown curve of the ceramide bilayer shows a stable plateau, illustrating the dense organization of ceramide molecules that contribute to the structural integrity of the bilayer. In contrast, the green curve for lignin derivatives reveals a more diffuse distribution, indicating their interaction across a wider range at the interface.

**Figure A4:**
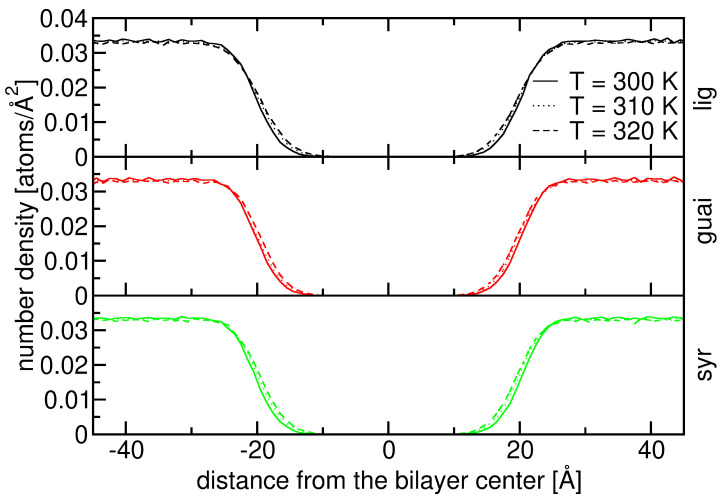
Number density distributions of water at varying temperatures, T = 300 K (solid line), T = 310 K (dotted line), and T = 320 K (dashed line), for systems containing guaiacyl units (red), syringyl units (green), and lignin derivatives (black). The x-axis represents the distance from the bilayer center, while the y-axis indicates the corresponding number density of water for each system.

**Figure A5:**
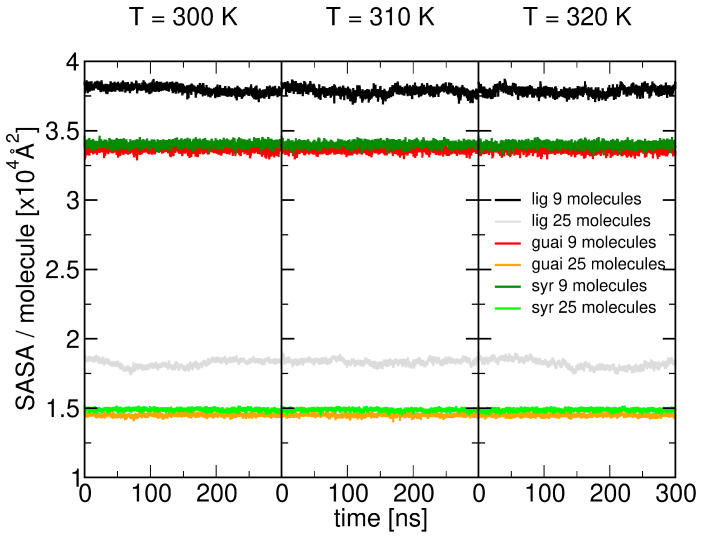
Solvent-accessible surface area (SASA)/molecule of guaiacyl units (red/orange), syringyl units (dark/light green), and lignin derivatives (black/grey). The systems contain two concentrations (9/25 molecules). Data are shown across different temperatures (300, 310, and 320 K), depicted in different columns.

**Figure A6:**
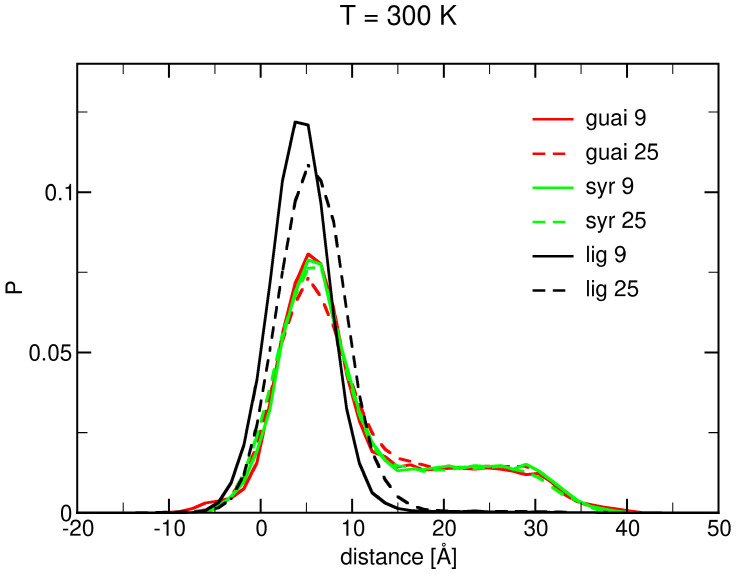
Distribution of COM distances of the guaiacyl units (red), syringyl units (green), and lignin derivatives (black) to the membrane at T = 300 K. The distance was calculated as the difference between the average z-coordinate of the nitrogen atoms and the center of mass of the guaiacyl unit, the syringyl unit, and the lignin derivative. Solid lines correspond to a concentration of 9 molecules, and dashed lines represent 25 molecules of each compound.

**Figure A7:**
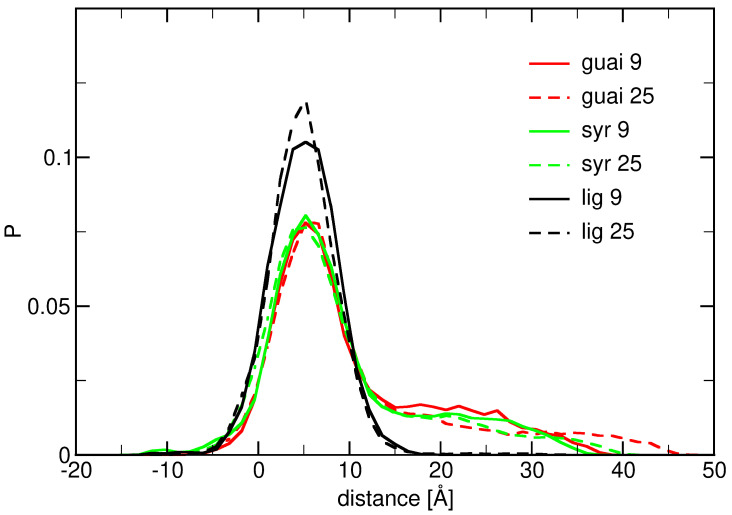
Distribution of COM distances of the guaiacyl units (red), syringyl units (green), and lignin derivatives (black) to the membrane at T = 320 K. The distance was calculated as the difference between the average z-coordinate of the nitrogen atoms and the center of mass of the guaiacyl unit, the syringyl unit, and the lignin derivative. Solid lines correspond to a concentration of 9 molecules, and dashed lines represent 25 molecules of each compound.

Figure A1 shows the number of hydrogen bonds formed between the lignin derivative and various components (A. ceramide bilayer; B. water; C. NF atom; D. OF atom; E. O1 atom; and F. O3 atom) plotted against the z-distance from the center of mass (COM) of the lignin derivative. The data in panel A shows a higher number of hydrogen bonds and frequency with the ceramide bilayer at the interface (between −5 Å and 0 Å), indicating that the lignin derivative interacts more strongly with the lipid membrane as it approaches the bilayer. Panel B reveals a higher interaction with water around 5 Å, but the highest frequency is maintained at the interface with the bilayer (−5 Å to 0 Å). The latter reflects the crucial importance of the hydration bilayer in lignin derivative’s interaction with the ceramide bilayer at the interface.

For the hydrogen bonds involving specific atoms (panels C to F), there is a noticeable variation across different z-distances. Panel C, showing the interaction with nitrogen (NF) atoms, indicates a higher concentration of bonds near z = 0, with a decrease as the lignin derivative moves further into the bilayer. Panels D, E, and F, which represent interactions with oxygen (OF, O1, and O3 atoms), demonstrate more localized peaks, particularly at certain z-distances where the lignin derivative is positioned near or within the lipid bilayer. These fluctuating hydrogen bond patterns illustrate how the lignin derivative’s interactions with water and lipid components change as it moves from a more aqueous environment into the hydrophobic region of the ceramide bilayer, emphasizing the dynamic nature of the system.

### 2.4. Factors Facilitating Lignin Insertion

Lignin insertion into skin lipid environments is primarily governed by its phenolic functional groups, which can interact with lipid components through weak, non-covalent associations. These interactions facilitate the initial adherence of lignin molecules to the lipid-rich stratum corneum, where hydrogen bonding and van der Waals forces stabilize their positioning. Additionally, lignin exhibits a pronounced tendency to self-associate, leading to clustering or aggregation on the skin surface [40]. Such phenomena are largely driven by hydrophobic interactions and intrinsic structural motifs within lignin, thereby modulating its spatial distribution and influencing its potential as a protective or antioxidant agent [59,63].

**Figure A8:**
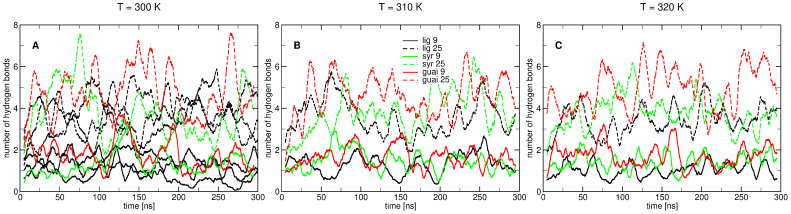
Time evolution of the number of intermolecular hydrogen bonds at T = (**A**). 300 K, (**B**). 310 K and (**C**). 320 K between lignin molecules and ceramide bilayer. Lines of the same color represent individual trajectories for two concentrations of lignin derivatives (black), with green indicating syringyl units and red indicating guaiacyl units. Solid lines correspond to 9 lignin derivatives, and dashed lines represent 25 lignin derivatives.

Hydrogen bonding plays a central role in regulating lignin–ceramide interactions, with molecular architecture exerting significant influence. Simulations performed at different temperatures revealed that monomeric syringyl and guaiacyl units formed a higher number of hydrogen bonds with the ceramide bilayer compared to trimeric derivatives (see Figure 6). This enhanced hydrogen-bonding capacity of monomers is due to their accessible hydroxyl groups and reduced steric hindrance, which promote more favorable orientations within the ceramide bilayer. In contrast, the trimeric lignin structures displayed steric constraints that limited efficient hydrogen bond formation, underscoring the importance of molecular size and conformational flexibility in governing the interaction strength between lignin derivatives and ceramide lipid bilayers (see Figure A8).

Furthermore, concentration-dependent effects were observed for all simulated temperatures (300–320 K), with systems containing higher lignin content exhibiting an increased number of hydrogen bonds over time (see Figure A8). Notably, guaiacyl units consistently outperformed syringyl units in hydrogen-bonding interactions with the ceramide bilayer, irrespective of temperature (see Figure 6). This disparity arises from steric hindrance imposed by the two methoxy groups of syringyl units, which limit the accessibility of hydroxyl groups for hydrogen bonding, whereas guaiacyl units present a more favorable orientation and bonding potential [60]. Collectively, these findings highlight the interplay of molecular structure, concentration, and temperature in dictating lignin–lipid interactions, thereby shaping the overall efficiency of lignin insertion into skin barrier systems.

**Figure A9:**
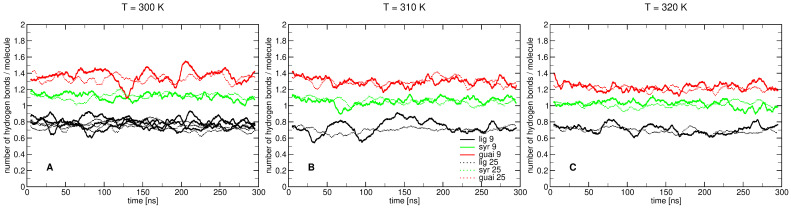
Time evolution of the number of intermolecular hydrogen bonds at T = (**A**). 300 K, (**B**). 310 K and (**C**). 320 K between lignin molecules and water. Lines of the same color represent individual trajectories for two concentrations of lignin derivatives (black), with green indicating syringyl units and red indicating guaiacyl units. Solid lines correspond to 9 lignin derivatives, and dashed lines represent 25 lignin derivatives.

The time evolution of the number of intermolecular hydrogen bonds formed between lignin derivatives and water at different temperatures (300, 310, and 320 K) reveals distinct differences depending on lignin subunit type and concentration (see Figure A9). Across all temperatures, guaiacyl units consistently form more hydrogen bonds with water than syringyl units, with average values near 1.4 bonds per molecule for syringyl compared to 1.2 for guaiacyl. The difference is given by the hindrance coming from the two methoxy groups of syringyl, as detailed above [60].

The influence of lignin concentration (9 vs. 25 molecules) on hydrogen bonding is relatively modest. The dashed lines (25 molecules) largely overlap with their solid-line counterparts (9 molecules), suggesting that intermolecular competition among lignin molecules does not substantially alter their ability to interact with water. Instead, the chemical nature of the lignin monomer type appears to be the dominant factor controlling hydrogen bond formation.

Temperature had only a slight impact on hydrogen-bonding dynamics. A minor decrease in the average number of hydrogen bonds per molecule is observed with increasing temperature, consistent with weaker hydrogen-bonding interactions at elevated thermal conditions. Nonetheless, the hydrogen-bonding patterns remain stable over the 300 ns trajectories at all three temperatures, indicating persistent lignin–water interactions (see Figure A9).

**Figure 8:**
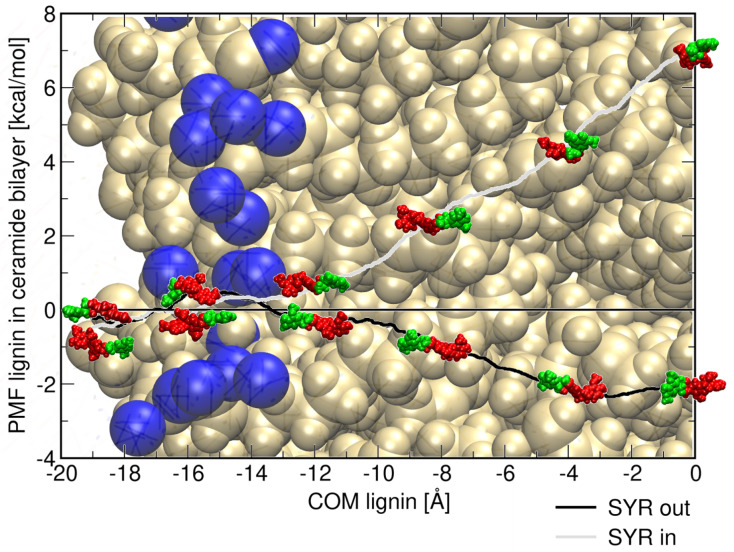
Potential of mean force (PMF) profiles of lignin derivatives inserted into the ceramide bilayer. PMF SYR in/down corresponds to the syringyl molecule head penetrating into the bilayer, while PMF SYR out/up corresponds to the guaiacyl molecule head inserted into the bilayer. Insets show representative snapshots of the molecules at different stages of insertion, with guaiacyl units depicted in red and syringyl units in green. For clarity, water molecules and ions have been omitted. The ceramide bilayer is shown in van der Waals (vdW) representation, with nitrogen atoms highlighted as blue spheres. These profiles and snapshots provide insight into the relative energetics and orientations of lignin derivatives within the ceramide bilayer.

The potential of mean force profiles (PMFs) along the normal to the ceramide bilayer *z* presented in Figure 8 provide quantitative insights into the free energy landscape governing the insertion of lignin derivatives into ceramide bilayers. Both PMFs present an almost flat energy surface up to 8 Åof insertion (up to −12 Å) into the ceramide bilayer. This result is consistent with the insertions of the lignin derivative observed in the unbiased molecular dynamics simulations.

The PMF profile corresponding to the insertion of the syringyl head (SYR in/down) reveals that the free energy increases progressively as the molecule penetrates deeper into the hydrophobic core of the bilayer. This indicates the presence of a substantial energetic barrier, consistent with the unfavorable positioning of SYR’s polar groups within the membrane interior. The rise in free energy near the bilayer center suggests that full insertion of the syringyl unit is energetically costly, limiting its ability to penetrate beyond the interfacial region. Consequently, syringyl-rich derivatives are expected to preferentially localize near the membrane surface, where they can maximize interactions with lipid headgroups while avoiding the hydrophobic core.

In contrast, the guaiacyl head insertion (SYR out/up) exhibits a very distinct PMF profile, characterized by an energy minimum localized at intermediate depths (at about 4 Å from the ceramide bilayer center), suggesting the stabilization of lignin derivatives with COM around that position. Although the overall free energy still rises toward the membrane center, guaiacyl units encounter a much lower energetic penalty compared to syringyl units, enabling deeper penetration into the ceramide bilayer. This differential behavior highlights the amphiphilic character of guaiacyl moieties, which are more adaptable to both polar and nonpolar environments. These results demonstrate that the compositional balance of syringyl and guaiacyl units critically influences the free energy landscape of lignin–ceramide interaction along the normal to the membrane, thereby shaping the extent, orientation, and stability of lignin insertion into the ceramide bilayer.

### 2.5. Role of Surface Clusters

The formation of surface clusters represents a critical aspect of molecular organization at the skin interface, influencing both structural and protective functions. In particular, clusters of lignin and its derivatives can act as a physical barrier, attenuating the penetration of ultraviolet (UV) radiation and thereby contributing to photoprotection [64,65]. Beyond this shielding effect, the clustering behavior of lignin-related molecules provides valuable information on their capacity to interact with lipid bilayers, an essential factor in understanding their role in maintaining skin barrier integrity.

**Figure 9:**
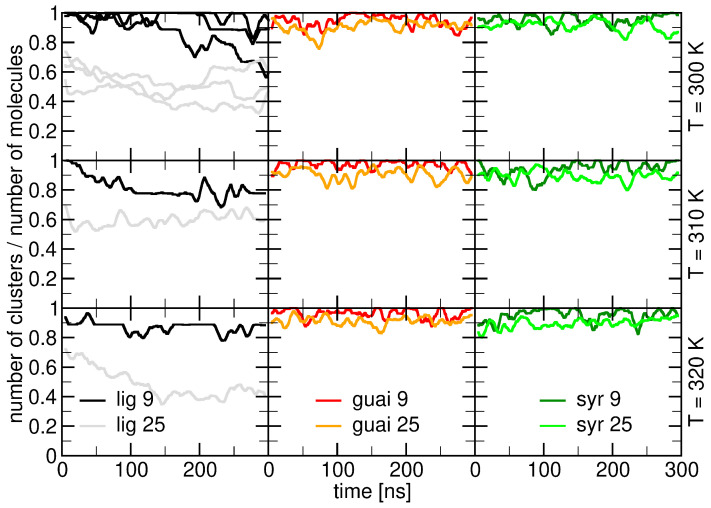
Time evolution of the number of clusters per total of number of lignin molecules for the guaiacyl units (red), syringyl units (green), and lignin derivatives (black) extracted from each simulation (represented by a line) plotted in a temperature-dependent manner (top row T = 300 K, middle row T = 310 K, and bottow row T = 320 K). The X axis represents simulation time (ns), and the Y-axis shows the cluster count, defined as the number of clusters containing the respective component per molecule of that component (i.e., clusters per guaiacyl molecule, per syringyl molecule, or per lignin-derivative molecule). Dark colors correspond to a molecular concentration of 9 molecules of each compound, while light colored lines correspond to 25 molecules of each compound.

The quantitative analysis of cluster formation, illustrated in Figure 9, offers deeper insights into their interaction dynamics. Specifically, our results highlight how guaiacyl, syringyl, and lignin derivatives associate with a ceramide bilayer under varying thermal conditions (T = 300 K, 310 K, and 320 K). The comparison between simulations with different molecular concentrations—9 versus 25 molecules per system, depicted by dark- and light-colored lines, respectively—enables the assessment of how both temperature and concentration modulate cluster stability and prevalence, as described below.

The temporal evolution of cluster formation across the studied temperatures (300 K, 310 K, and 320 K) demonstrates that clustering behavior remains largely stable within this thermal window. In the case of lignin derivatives, elevated temperatures do not significantly disrupt molecular self-association. This temperature invariance suggests that the interaction patterns of lignin derivatives with the ceramide bilayer are preserved under physiologically relevant conditions. Such robustness highlights the potential of lignin derivatives for cosmetic applications, as they can maintain structural and functional integrity of skin-like membranes even under variable thermal environments.

A comparison between the three compound types highlights clear differences in their clustering propensity (see Figure 9). Lignin derivatives exhibit consistently higher levels of cluster formation compared to guaiacyl and syringyl units, regardless of concentration or temperature. This trend suggests that the chemical complexity and multifunctional character of lignin derivatives promote stronger intermolecular associations, likely through additional noncovalent interactions (e.g., hydrogen bonding). In contrast, the monomeric guaiacyl and syringyl units show more limited aggregation, reflecting their reduced ability to form stable cluster networks at the bilayer interface.

The role of concentration further underscores the importance of molecular density in promoting clustering. Simulations with 25 molecules (light-colored lines) consistently display reduced normalized cluster numbers per molecule compared to those with 9 molecules (dark-colored lines), consistent with the expectation that higher concentrations increase the likelihood of molecular contacts and favor the formation of larger, fewer clusters (see Figure 9). This concentration-dependent effect is especially pronounced for lignin derivatives, where clustering efficiency improves markedly at elevated molecular densities. Taken together, these results demonstrate that clustering dynamics are robust against temperature variation, enhanced by molecular complexity, and strongly modulated by concentration.

In summary, the data presented in all three figures illustrate that lignin derivatives exhibit a greater propensity for clustering compared to syringyl and guaiacyl units, with this behavior being modulated by both temperature and concentration. At lower concentrations, lignin derivatives form more clusters than syringyl and guaiacyl compounds. As the concentration increases, the number of clusters decreases for all compounds, with the lignin derivatives showing a more stable clustering pattern over time. These findings emphasize the importance of both the molecular composition and concentration in determining the clustering behavior of lignin derivatives when interacting with biological membranes.

**Figure 10:**
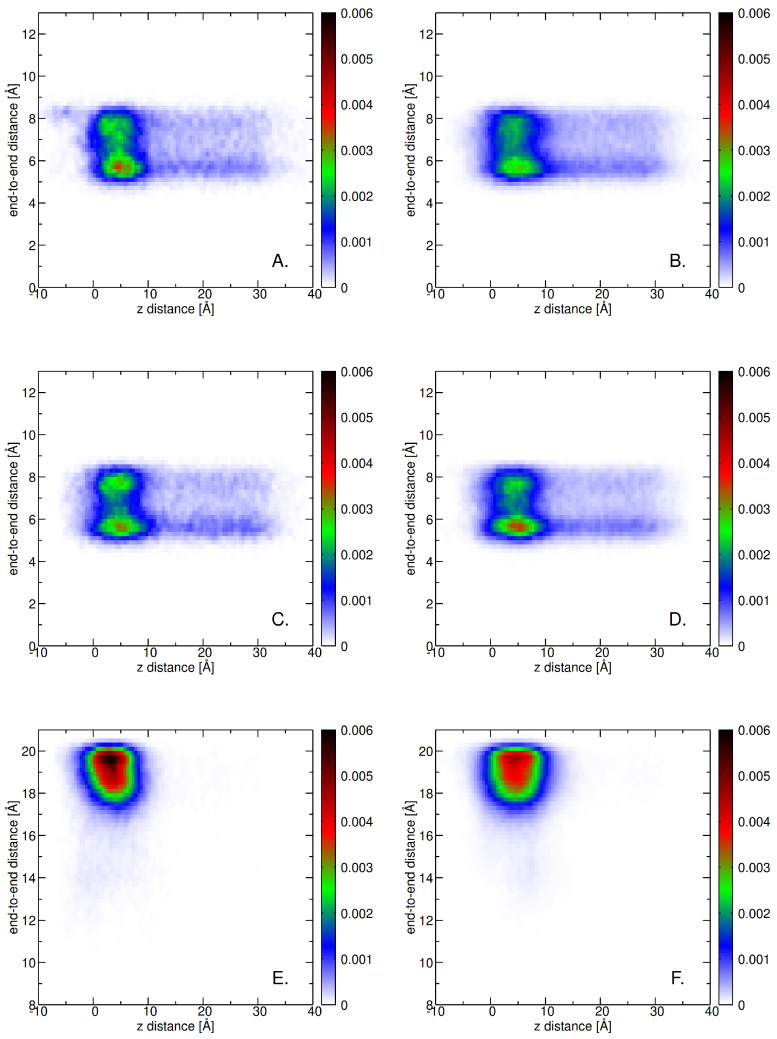
End-to-end versus *z*-distance distributions of guaiacyl units (**A**,**B**), syringyl units (**C**,**D**), and lignin derivatives (**E**,**F**) relative to the membrane at a temperature of T=300 K. The left column (**A**,**C**,**E**) depicts the distribution for a system with 9 molecules, while the right column (**B**,**D**,**F**) illustrates the distribution for a system containing 25 molecules. The color map indicates the normalized frequency of occurrence, providing a visual representation of how these lignin-derived units are spatially distributed in relation to the membrane interface. The end-to-end distance is calculated as the distance between the terminal atoms of each unit for guaiacyl (atoms O4 and O9), for syringyl (also atoms O4 and O9), and for lignin derivatives (between residue 1, atom O4, and residue 3, atom O4). The *z*-distance reflects the vertical position of these atoms with respect to the membrane surface, allowing for assessment of their proximity to the lipid bilayer.

Figure 10 displays the center-of-mass (COM) distance distributions in terms of end-to-end distance versus the *z*-axis COM distance of guaiacyl units from the membrane at 300 K. The left column corresponds to systems containing 9 molecules, and the right column to those with 25 molecules. At this temperature, the guaiacyl units are primarily localized at a *z*-distance near 0–10 Å, indicating strong association with the membrane surface. The quasi-continuous variations of the end-to-end distance (5 Å–8.5 Å) corresponds to the fluctuations of the tail attached to the ring, with dominant conformations being a more compact one at around 5.5 Å and a more extended conformation close to 8 Å. The syringyl units (second row) have stronger propensity for the compact and extended conformations than guaiacyl. The bottom row shows that the lignin derivative is much more rigid than the other two units (in spite of being a trimer).

**Figure A10:**
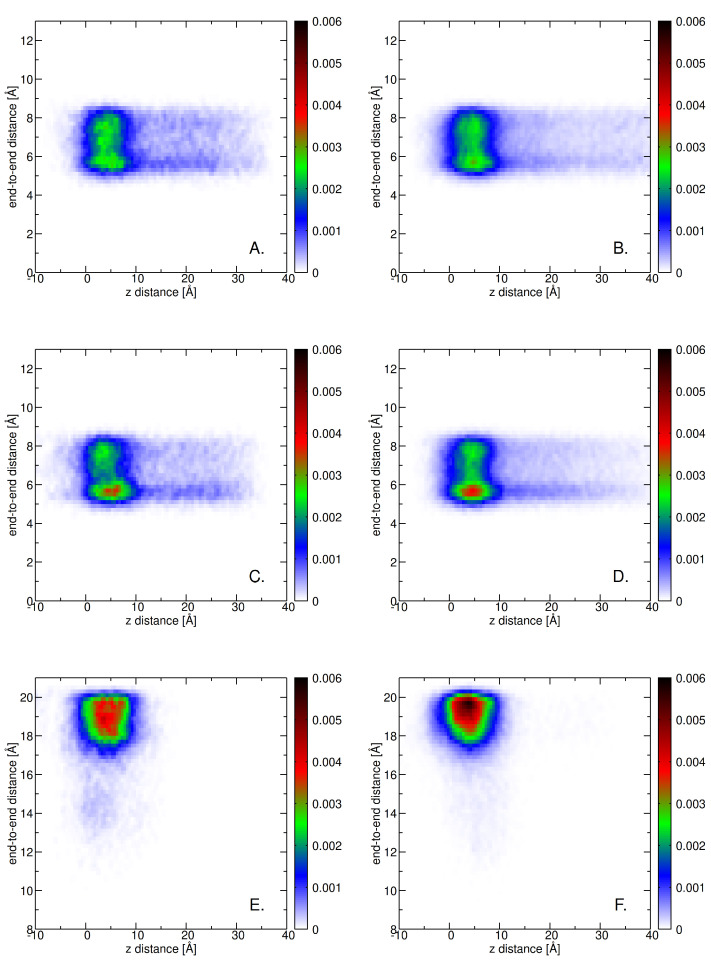
End-to-end versus *z*-distance distributions of guaiacyl units (**A**,**B**), syringyl units (**C**,**D**), and lignin derivatives (**E**,**F**) relative to the membrane at a temperature of T=310 K. The left column (**A**,**C**,**E**) depicts the distribution for a system with 9 molecules, while the right column (**B**,**D**,**F**) illustrates the distribution for a system containing 25 molecules. The color map indicates the normalized frequency of occurrence, providing a visual representation of how these lignin-derived units are spatially distributed in relation to the membrane interface. The end-to-end distance is calculated as the distance between the terminal atoms of each unit: for guaiacyl (atoms O4 and O9), for syringyl (also atoms O4 and O9), and for lignin derivatives (between residue 1, atom O4, and residue 3, atom O4). The *z*-distance reflects the vertical position of these atoms with respect to the membrane surface, allowing for assessment of their proximity to the lipid bilayer.

**Figure A11:**
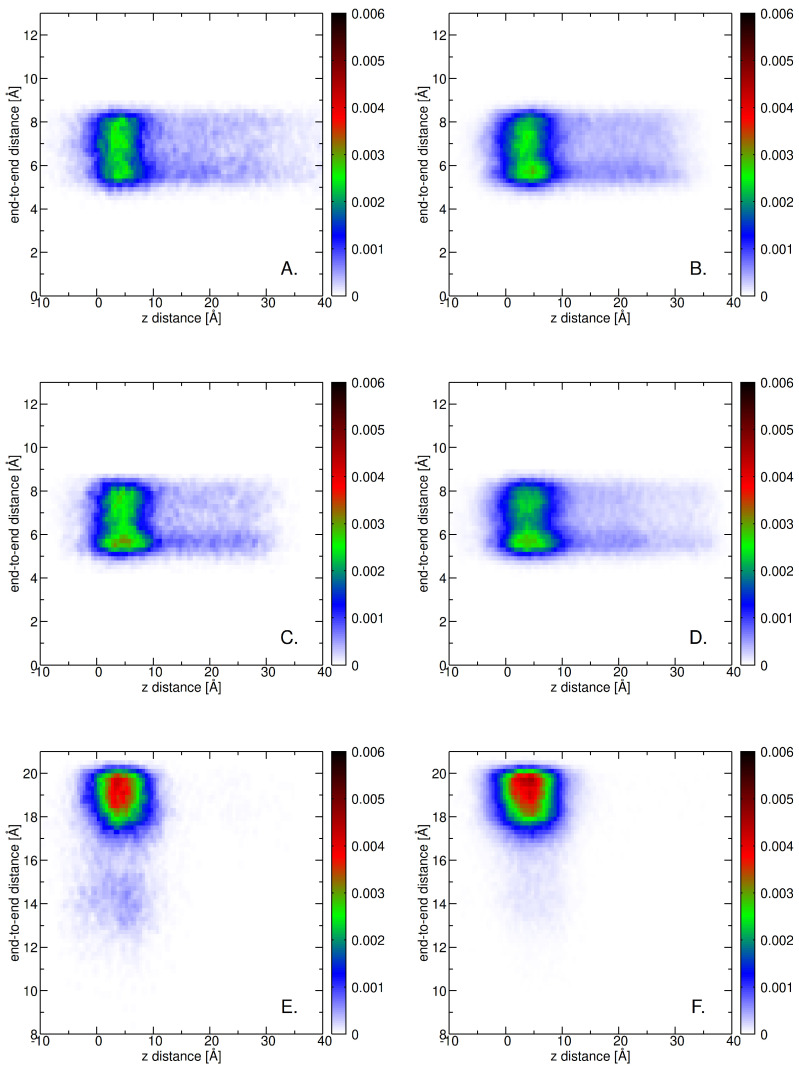
End-to-end versus *z*-distance distributions of guaiacyl units (**A**,**B**), syringyl units (**C**,**D**), and lignin derivatives (**E**,**F**) relative to the membrane at a temperature of T=320 K. The left column (**A**,**C**,**E**) depicts the distribution for a system with 9 molecules, while the right column (**B**,**D**,**F**) illustrates the distribution for a system containing 25 molecules. The color map indicates the normalized frequency of occurrence, providing a visual representation of how these lignin-derived units are spatially distributed in relation to the membrane interface. The end-to-end distance is calculated as the distance between the terminal atoms of each unit: for guaiacyl (atoms O4 and O9), for syringyl (also atoms O4 and O9), and for lignin derivatives (between residue 1, atom O4, and residue 3, atom O4). The *z*-distance reflects the vertical position of these atoms with respect to the membrane surface, allowing for assessment of their proximity to the lipid bilayer.

Our MD simulations have shown that guaiacyl, syringyl, and lignin derivatives exhibit similar behavior with the increase in temperature (see Figure A10 and Figure A11). The broader range of end-to-end distances (below 17 Å) is (slightly) present only at low concentrations. This further indicates that even at high temperatures, the lignin derivatives are adopting more extended conformations as opposed to individual monomeric units (see Figure A11).

**Figure A12:**
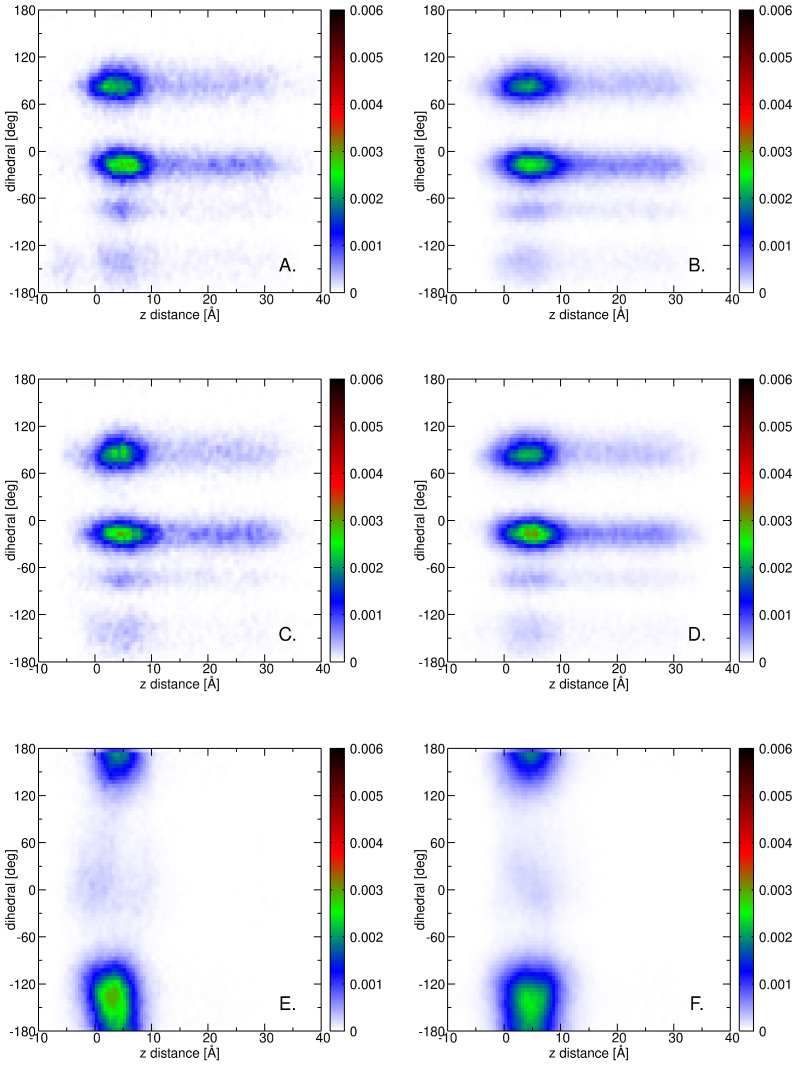
Dihedral versus *z*-distance distributions of guaiacyl units (**A**,**B**), syringyl units (**C**,**D**), and lignin derivatives (**E**,**F**) relative to the membrane at a temperature of T=300 K. The left column (**A**,**C**,**E**) depicts the distribution for a system with 9 molecules, while the right column (**B**,**D**,**F**) illustrates the distribution for a system containing 25 molecules. The color map indicates the normalized frequency of occurrence, providing a visual representation of how these lignin-derived units are spatially distributed in relation to the membrane interface. The dihedral angle, which reflects the rotation about the bond angles involving terminal atoms, is calculated for: guaiacyl units (from atoms O4, C7, and O9), syringyl units (also using O4, C7, and O9), and lignin derivatives (between the terminal atom O4 of residue 1, L1 and residue 2’s atom O4, and residue 3’s atom O4).

The distributions in Figure A12 illustrate the conformational flexibility and spatial positioning of guaiacyl units (A, B), syringyl units (C, D), and lignin derivatives (E, F) relative to the membrane interface at T=300 K. The end-to-end distance, plotted against the *z*-distance, reveals distinct differences between monomeric units and lignin derivatives. Guaiacyl and syringyl moieties (panels A–D) display localized distributions with preferred dihedral angles, indicating relatively constrained conformational states when interacting with the bilayer. These preferred conformations are centered around two main dihedral orientations (one between 0 and 30 degrees and a second between 75 and 105 degrees), consistent with the two rings’ tail flexibility. In contrast, lignin derivatives (panels E, F) exhibit a broader conformational space, with distributions extending across one restricted dihedral range (from 150 degrees up to −90 degrees), consistent with their greater structural rigidity (see Figure 10).

**Figure A13:**
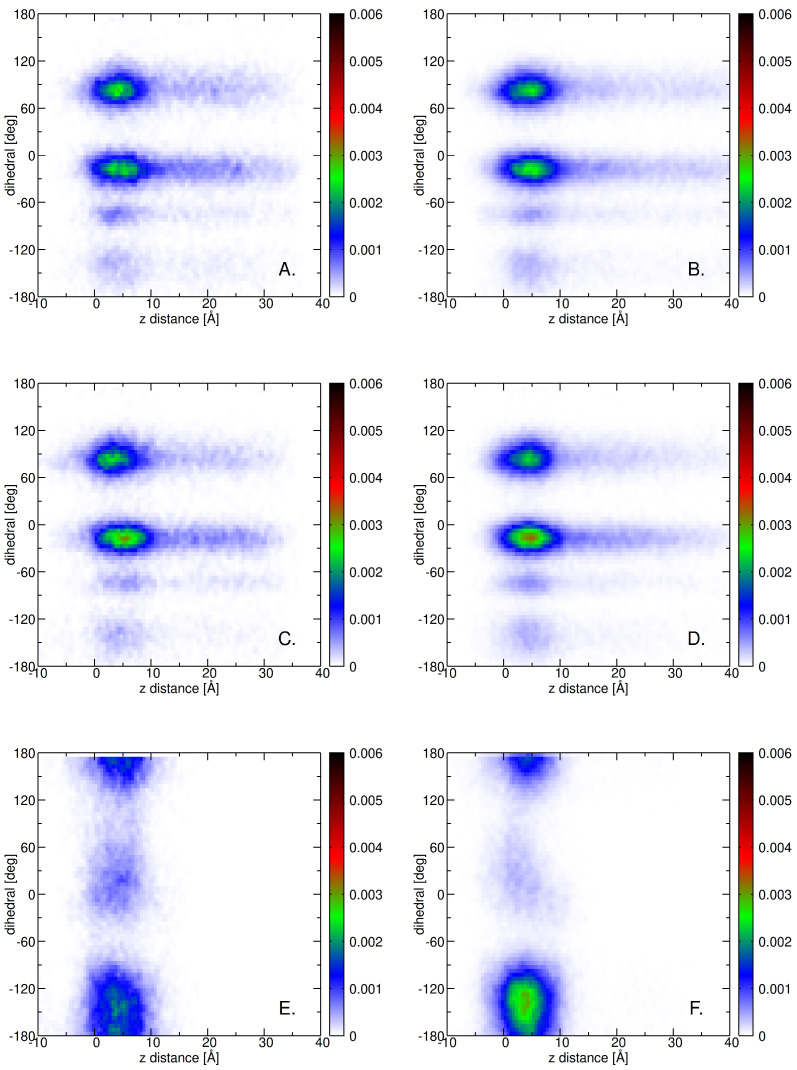
Dihedral versus *z*-distance distributions of guaiacyl units (**A**,**B**), syringyl units (**C**,**D**), and lignin derivatives (**E**,**F**) relative to the membrane at a temperature of T=310 K. The left column (**A**,**C**,**E**) depicts the distribution for a system with 9 molecules, while the right column (**B**,**D**,**F**) illustrates the distribution for a system containing 25 molecules. The color map indicates the normalized frequency of occurrence, providing a visual representation of how these lignin-derived units are spatially distributed in relation to the membrane interface. The dihedral angle, which reflects the rotation about the bond angles involving terminal atoms, is calculated for: guaiacyl units (from atoms O4, C7, and O9), syringyl units (also using O4, C7, and O9), and lignin derivatives (between the terminal atom O4 of residue 1, L1 and residue 2’s atom O4, and residue 3’s atom O4).

**Figure A14:**
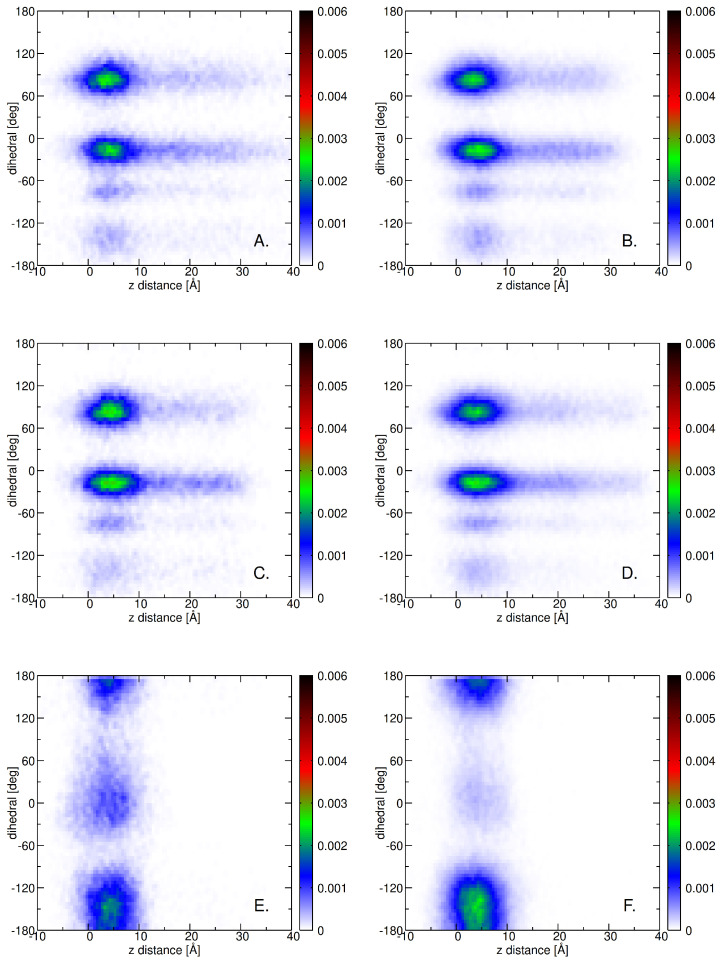
Dihedral versus *z*-distance distributions of guaiacyl units (**A**,**B**), syringyl units (**C**,**D**), and lignin derivatives (**E**,**F**) relative to the membrane at a temperature of T=320 K. The left column (**A**,**C**,**E**) depicts the distribution for a system with 9 molecules, while the right column (**B**,**D**,**F**) illustrates the distribution for a system containing 25 molecules. The color map indicates the normalized frequency of occurrence, providing a visual representation of how these lignin-derived units are spatially distributed in relation to the membrane interface. The dihedral angle, which reflects the rotation about the bond angles involving terminal atoms, is calculated for guaiacyl units (from atoms O4, C7, and O9), syringyl units (also using O4, C7, and O9), and lignin derivatives (between the terminal atom O4 of residue 1, L1 and residue 2’s atom O4, and residue 3’s atom O4).

Interestingly, simulations conducted at higher temperatures (T=310 K and T=320 K, see Figure A13 and Figure A14) reveal similar trends in conformational behavior and spatial positioning of the lignin derivatives. However, the higher concentration preserves best their described behavior pattern, which suggests that enhanced clustering conserves internal degrees of freedom ranges (such as dihedral angle) even at high temperatures (see panels E and F in Figure A13 and Figure A14). This suggests that the observed interactions and conformational preferences are robust to moderate thermal fluctuations, reinforcing the stability of lignin–bilayer interactions under physiologically relevant conditions.

## 3. Discussion

The current study sheds new light on the molecular mechanisms that dictate the interactions of lignin derivatives with skin-like ceramide bilayers. Utilizing molecular dynamics simulations, we revealed that the insertion behavior of syringyl and guaiacyl units is significantly influenced by their molecular structure, concentration, and temperature. Smaller lignin derivatives exhibited rapid and spontaneous insertion events, driven by a transition from a solvated environment, characterized by diminishing hydrogen bonds with water, to one stabilized by increased hydrogen bonding with lipid headgroups. Importantly, guaiacyl units consistently formed a greater number of hydrogen bonds with ceramide bilayer than syringyl units across all tested conditions, indicating that the specific chemical functionality of these lignin subunits is crucial for stabilizing bilayer interactions.

The influence of structure and temperature on lignin behavior within lipid bilayer systems is complex. Elevated temperatures increase lignin’s conformational flexibility and solvent exposure, as indicated by rising solvent-accessible surface area values, reflecting more dynamic and accessible molecular states. Chemical modifications, concentration, and molecular architecture—particularly differences between guaiacyl and syringyl units—affect lignin aggregation and membrane interactions. Larger or chemically altered lignin derivatives tend to aggregate more and remain on the membrane surface. Molecular dynamics simulations with extended trajectories across various temperatures and concentrations show that higher temperatures promote closer association and more compact lignin arrangements within the bilayer, affecting their spatial distribution and mobility. Overall, these findings demonstrate that environmental conditions and lignin structure significantly influence their structural and dynamic behavior in membrane systems.

Lignin molecules display a strong tendency to rapidly adhere to skin-like lipid surfaces through physical interactions such as hydrogen bonding, which facilitate the initial attachment. Quantitative analyses show that lignin derivatives preferentially localize near the lipid–water interface, with higher concentrations fostering more ordered packing and larger clusters, especially at lower temperatures. Temperature influences the stability of these clusters; increased thermal motion at elevated temperatures reduces the number of clusters but still favors the formation of more stable aggregates of larger lignin derivatives compared to the small syringyl and guaiacyl subunits. Overall, the structural features and concentration of lignin determine its clustering behavior, impacting membrane properties and surface barrier functions.

Hydrogen bonding patterns are essential to the lignin–lipid interactions. Our data indicates that lignin monomers, particularly those containing syringyl and guaiacyl groups, form extensive hydrogen bonds with ceramide bilayers. The number of these bonds increases with concentration and is higher for monomeric forms due to reduced steric hindrance. Guaiacyl units tend to establish more hydrogen bonds than syringyl units, due to the increased accessibility of their functional groups, resulting in stronger and more numerous hydrogen bonding interactions. Temperature modulates these interactions by affecting hydrogen bond dynamics; nonetheless, higher temperatures only slightly decrease hydrogen bond numbers and do not completely eliminate the interactions. The distribution and stability of hydrogen bonds, along with conformational analyses, reveal that lignin’s molecular architecture—specifically the presence and accessibility of syringyl and guaiacyl functional groups—directly determines its capacity to form stable and extensive hydrogen bonding networks with ceramide bilayers. This heightened affinity, driven by accessible hydroxyl groups and favorable molecular conformations, enhances lignin’s ability to embed within and interact strongly with membrane surfaces.

The free energy profiles derived from PMF calculations elucidate the energetics of lignin–ceramide bilayer interactions. Guaiacyl units show a stronger affinity for membrane interfaces, characterized by more hydrogen bonds and deeper penetration into the membrane, reflected in lower free energy barriers for insertion. Conversely, syringyl units face substantial energetic barriers to full insertion, favoring surface localization due to their polar nature and less favorable hydrophobic interactions. These profiles, supported by structural snapshots, demonstrate that lignin derivatives can spontaneously insert into membranes driven by hydrogen bonding and amphiphilic interactions. However, their depth of insertion and stability are highly dependent on their chemical composition, influencing membrane integrity, permeability, and protective functions within biological or bioengineering contexts.

Collectively, these insights enhance our understanding of how molecular size, concentration, and temperature collaboratively govern lignin–bilayer interactions. The distinct roles of syringyl and guaiacyl units demonstrate a tunable balance between structural rigidity and conformational flexibility, which opens opportunities for the design of lignin-based compounds aimed at membrane-specific interactions. Furthermore, the observed clustering at the membrane interface suggests potential strategies for enhancing drug delivery or skin barrier protection through lignin-derived formulations. By integrating structural, energetic, and thermodynamic perspectives, this study lays the groundwork for the rational engineering of lignin derivatives as natural, sustainable agents in dermatological applications.

## 4. Materials and Methods

### 4.1. Molecular Dynamics

**Figure 11:**
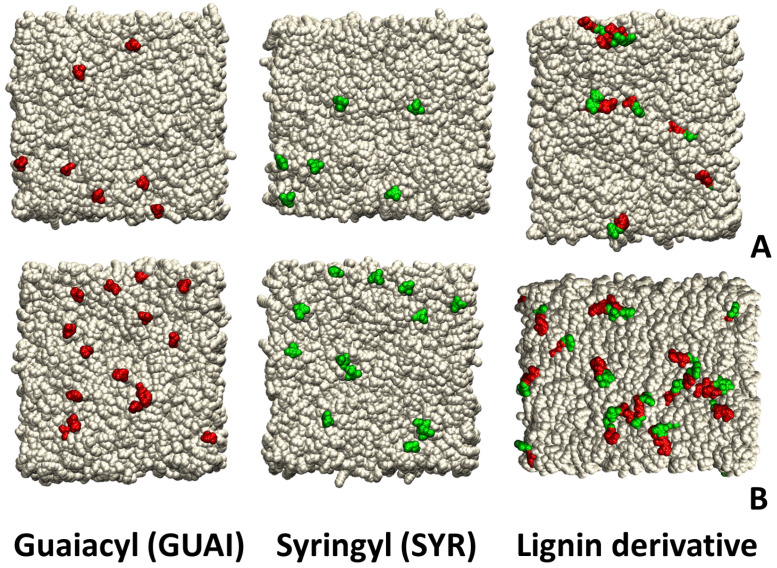
Snapshots depicting the final configurations of systems studied. The ceramide bilayer is shown in cream using a van der Waals (vdW) representation, highlighting the bilayer’s structural integrity. (**A**) The system comprising the ceramide bilayer with nine guaiacyl, syringyl, or lignin derivatives is presented, with guaiacyl and syringyl depicted in red and green using vdW representation to emphasize their spatial distribution and interactions within the bilayer environment. (**B**) The system containing the ceramide bilayer and twenty-five guaiacyl, syringyl, or lignin derivatives is illustrated, demonstrating increased lignin incorporation and effects on bilayer organization.

All the input files required to build the simplified model for skin membranes, namely a ceramide bilayer (CER181 18:1/18:1) for molecular dynamics simulations, were generated using CHARMM-GUI [66,67]. The ceramide bilayer system was constructed as a rectangular box (see Figure 11) containing a total of 800 lipids (400 per leaflet) (see Table 1). The lignin derivatives were placed on a 3 × 3, respectively, 5 × 5 grid of molecules, in which the center of mass for each molecule was positioned at a grid point. Fixed-charge MD simulations were performed at neutral pH and 0.15 M ionic strength. The systems were solvated and neutralized by sodium and chloride ions to achieve a 0.15 M salt concentration.

All MD simulations were performed with the NAMD 2.13 [68] molecular dynamics software package using the CHARMm C36 force field for lipids [69] and the CHARMm All-Atom Additive Force Field for sphingomyelin [70]. The force field developed by Vermaas et al. [18] was used for small lignin derivative molecules. A step cycle of 20 and a time step of 2 fs was used in conjunction with the SHAKE algorithm [71,72] to constrain the covalent bonds involving hydrogen atoms. Long-range electrostatic interactions were calculated using the particle mesh Ewald method [73] with a spacing of 1 Å and a cutoff distance of 12 Å. To control the pressure in our simulations, we utilized a semi-isotropic approach. In all simulations, a Langevin thermostat [74] with a damping coefficient of 1 ps^−1^ to maintain a constant temperature of 300/310/320 K was used. The pressure was controlled using a Langevin piston [75] at 1 atm.

The following protocol was used in all simulations: (a) The systems were minimized for 10000 steps with the following atoms harmonically restrained: the oxygen in the bilayer (k = 2.5 kcal mol^−1^Å^−2^), water oxygen and ions (k = 0.1 kcal mol^−1^Å^−2^). (b) A 150 ps warmup from 0 K to 300 K was performed, with an increase of 3 K in temperature at every ps. The following atoms were harmonically restrained: the oxygen in the bilayer (k = 1 kcal mol^−1^Å^−2^), water oxygen and ions (k = 0.1 kcal mol^−1^Å^−2^). (c) A 350 ps constrained equilibration was performed at 300/310/320 K, along the normal to the membrane direction only, with the constrained oxygen in the bilayer (k = 0.1 kcal mol^−1^Å^−2^). (d) For each simulation, a production run of 300 ns was performed for the three corresponding temperatures (300/310/320 K). The thermal conditions were extended to include 300 K, 310 K, and 320 K to span storage, handling, and application scenarios for lignin derivative formulations. In addition to reproducing physiological skin temperature (310 K), the inclusion of 320 K allows assessment of temperature-induced changes in lignin–membrane interactions under elevated temperature conditions that may occur during heat exposure or high-temperature storage. Moreover, lignin’s UV-absorbing properties may confer photoprotective benefits in cosmetic products, motivating the exploration of these interactions across the temperature range [76]. All the snapshots from the simulations have been extracted using the VMD package [77].

### 4.2. Potential of Mean Force Calculations

The FR method proposed by Kosztin et al. [78] is a well-established non-equilibrium approach that can be easily implemented to compute diffusion coefficients and potential of mean forces (PMFs) along reaction coordinate(s) in order to attain the molecular adsorption to a lipid bilayer [79], predict molecular permeability [80,81,82] and protein complex oligomerization [83], and compute water/ion conduction in channels [84,85]. By successfully exploiting fluctuation theorem-based [86] non-equilibrium simulations, the FR method was also used to develop novel approaches [87,88,89,90] and to connect experimental results to simulations [91]. Here, we used the FR method to determine the PMF profile of the lignin derivative’s (LD) center-of-mass (COM) along the normal to the (ceramide skin-like) membrane model (*z*).

Assuming overdamped Brownian dynamics and the stiff spring approximation, the PMF can be estimated from the average forward (F) and reverse (R) works (W) using the following equation [78]:

(1) $$\Delta U\left(z\right)=0.5\left(\langle W_{F}\rangle -\langle W_{R}\rangle \right)$$

In order to determine the mean values of the F and R works, we used several pulls starting from a variety of initial lignin derivative conformations, obtained from fast initial constant velocity pulls. The advantage of using the FR method for calculating ΔU(z) is two-fold: (i) it uses a relatively small number of fast non-equilibrium F and R pulls, and (ii) it uses only the mean values of the works along these F and R pulls. For the constant velocity-steered molecular dynamics simulations required for obtaining the $<W_{F}>$ and $<W_{R}>$ works of lignin derivatives’ COMs along the normal to the membrane *z*, we used v(z)=0.1 Å/ns from the center of the ceramide bilayer (z=0 Å) to its surface (z=19 Å). In our PMF calculations, we used the hypothesis that the F/R pulls were performed under the condition that the lignin derivatives are single molecules. This is a reasonable approximation, supported by the low mol:mol ratio of the lignin derivatives to the ceramide lipids (9:800 ≈ 1:89 and 25:800 = 1:32 for the two systems, respectively).

The z-dependent works, corresponding to each non-equilibrium pull ($W_{Fi}\left(z\right)$ and $W_{Rj}\left(z\right)$), required to calculate the average work values by the non-equilibrium PMF calculations (as described in ref [78]), were calculated on the fly in NAMD [68] during the SMD simulations using an in-house TCL script *hgp-pull.tcl* (proposed by Kosztin et al. in ref [78], available online at https://github.com/lorantj/hgp-pull/tree/main/common/hgp-pull.tcl, accessed on 7 October 2025).

## 5. Conclusions

This work highlights the critical influence of molecular size, concentration, and thermal conditions on the dynamics of lignin derivatives interactions with skin-mimicking membranes. The distinct behaviors of lignin derivatives—ranging from rapid insertion and hydrogen bonding to clustering and surface localization—highlight their potential for tailored applications in skincare and transdermal delivery systems. The concentration influences surface clustering and membrane affinity, collectively shaping the protective and permeation-modulating properties of lignin-based compounds. The detailed insights into free energy landscapes and interaction mechanisms provided by molecular simulations facilitate the rational design of lignin derivatives with optimized membrane interactions, paving the way for sustainable, bio-based strategies in dermatological and therapeutic formulations and barrier enhancement technologies. In light of these findings, the design and engineering of mixed lignin derivatives will be crucial in tailoring their properties for complex skin-membrane model contexts. Future investigations should focus on optimizing the molecular architecture of lignin derivatives to achieve desired performance metrics, particularly concerning their affinity for skin membranes and their ability to modulate permeation rates effectively.

## Figures and Tables

**Table 1:** This table provides a detailed overview of the systems used in molecular dynamics simulations to investigate guaiacyl, syringyl, and lignin derivative molecules. It summarizes the simulation parameters across various systems, highlighting the types and quantities of molecules, temperature conditions, and the resulting system sizes. Specifically, the table includes systems with 9 and 25 molecules of guaiacyl and syringyl subunits, as well as a lignin derivative. All systems are simulated utilizing similar lipid and water compositions, with the total number of atoms varying slightly depending on the molecular composition.

System	Molecule	No. of Molecules	No. of Lipids	No. of Water Molecules	No. of Na^+^	No. of Cl^−^	Total No. of Atoms
**1**		0	800	39,910	113	113	163,585
**2**	guai	9	800	39,910	113	113	207,417
**3**	guai	25	800	39,910	113	113	207,881
**4**	syr	9	800	39,910	113	113	207,417
**5**	syr	25	800	39,910	113	113	20,7881
**6**	lig	9	800	39,910	113	113	209,031
**7**	lig	25	800	39,910	113	113	209,031

## Data Availability

The original contributions presented in this study are included in the article. Further inquiries can be directed to the corresponding author.
